# Exploration of human cerebrospinal fluid: A large proteome dataset revealed by trapped ion mobility time-of-flight mass spectrometry

**DOI:** 10.1016/j.dib.2020.105704

**Published:** 2020-05-16

**Authors:** Charlotte Macron, Regis Lavigne, Antonio Núñez Galindo, Michael Affolter, Charles Pineau, Loïc Dayon

**Affiliations:** a Proteomics, Nestlé Institute for Food Safety & Analytical Sciences, Nestlé Research, 1015 Lausanne, Switzerland; b Univ Rennes, Inserm, EHESP, Irset (Institut de recherche en santé, environnement et travail)-UMR_S 1085, 35042 Rennes cedex, France; c Protim, Univ Rennes, F-35042 Rennes, France; d Institut des Sciences et Ingénierie Chimiques, Ecole Polytechnique Fédérale de Lausanne (EPFL), 1015 Lausanne, Switzerland

**Keywords:** Cerebrospinal fluid, Large-scale proteome, Mass spectrometry, LC-MS/MS, Proteomics

## Abstract

Cerebrospinal fluid (CSF) is a biofluid in direct contact with the brain and as such constitutes a sample of choice in neurological disorder research, including neurodegenerative diseases such as Alzheimer or Parkinson. Human CSF has still been less studied using proteomic technologies compared to other biological fluids such as blood plasma or serum. In this work, a pool of “normal” human CSF samples was analysed using a shotgun proteomic workflow that combined removal of highly abundant proteins by immunoaffinity depletion and isoelectric focussing fractionation of tryptic peptides to alleviate the complexity of the biofluid. The resulting 24 fractions were analysed using liquid chromatography coupled to a high-resolution and high-accuracy timsTOF Pro mass spectrometer. This state-of-the-art mass spectrometry-based proteomic workflow allowed the identification of 3’174 proteins in CSF. The dataset reported herein completes the pool of the most comprehensive human CSF proteomes obtained so far. An overview of the identified proteins is provided based on gene ontology annotation. Mass and tandem mass spectra are made available as a possible starting point for further studies exploring the human CSF proteome.

Specifications table

**Table:** 

Subject	Proteomics
Specific subject area	Comprehensive proteome profiling of “normal” human cerebrospinal fluid (CSF) using mass spectrometry (MS).
Type of data	Liquid chromatograply tandem mass spectrometry (LC–MS/MS) data.
How data were acquired	LC-MS/MS acquisition on a nanoElute LC system coupled to a timsTOF Pro mass spectrometer.
Data format	Raw and processed.
Parameters for data collection	We re-analyzed samples previously analyzed in a report by Macron et *al.*[1]. A commercial pool of “normal” human CSF samples was prepared according to a previously published proteomic workflow [2,3], described in the following Method section. Sample fractionation was used.
Description of data collection	LC-MS/MS analyses of the resulting 24 fractions were performed using a nanoElute LC system, coupled to a timsTOF Pro mass spectrometer, to evaluate the instrumental performances for the proteomic profiling of CSF with respect to other LC-MS technologies [1]. Mass spectral data were searched using Mascot and X! Tandem search engines before being visualized and validated with the Scaffold software.
Data source location	Nestlé Research, 1015 Lausanne, Switzerland.
Data accessibility	Protein and peptide lists are provided in **Supplementary Table S1**. Repository name: ProteomeXchange Consortium. Data identification number: PXD018369.

## Value of the data

• A comprehensive proteomic profile of “normal” human CSF, among the largest reported so far using LC-MS/MS, is provided
• The data is useful for enhanced characterization and annotation of the human CSF proteome
• The data is valuable for the proteomic community for spectral library generation and as a starting point for clinical studies focussing on CSF and neurological disorders
• The data provides information for targeted protein/peptide assay development in human CSF

## 1 Data description

**Fig. 1:**
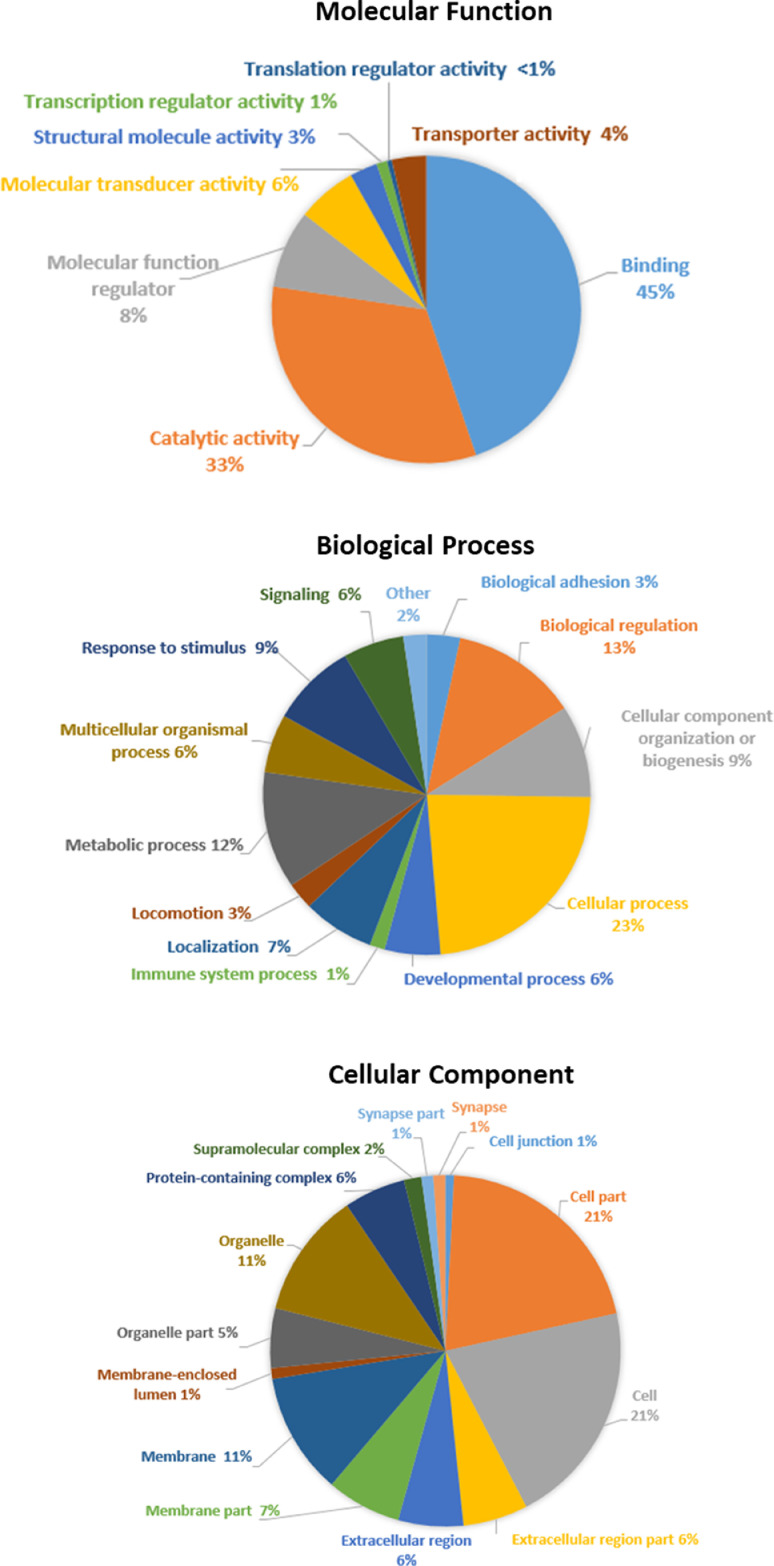
GO terms of the genes representative of the 3’174 proteins identified in the CSF dataset. The Panther software was used for the GO annotation on the three ontologies, (a) molecular function (b) biological process and (c) cellular component.

The dataset presented herein identified 3’174 proteins and their respective 25’227 peptides in “normal” CSF; protein and peptide lists are provided in **Supplementary Table S1**. The human CSF sample analyzed in this report was previously analyzed with different LC-MS/MS intrumentations to assess throughput and robustness of an automated pipeline for biomarker discovery [4] and to deeply charaterize the human CSF proteome in the quest of identificaton of missing proteins [1,5]. In the present work, the previously prepared sample was analyzed again using the recent timsTOF Pro mass spectrometer to evaluate its capabilities in terms of CSF proteome coverage. MS data were thus acquired by analysing CSF depleted from abundant proteins, after tryptic digestion and peptide fractionation, using a nanoElute LC system coupled to a timsTOF Pro mass spectrometer. MS raw files were then converted into peaklists with MSConvert and searched against the human UniProtKB/Swiss-Prot database using Mascot and X! Tandem. The Scaffold software, specifying a false discovery rate (FDR) of 1% at both protein and peptide level, and a one unique peptide criterion, was used to report protein identifications. Gene Ontology (GO) annotation was performed with the Panther software (Fig. 1). *Binding* and *Catalytic activity* represented 78% of the molecular functions. *Cellular process* was the most important biological process represented (*i.e.*, 23% of all genes); lastly, *Cell* and *Cell part* (21% each) were the major cellular components identified in this dataset.

A GO enrichment was also performed with Gorilla [6], to identify terms enriched in this “normal” human CSF sample with respect to the whole human proteome (Table 1). Terms relative to semaphorin/neuropilin/plexin, such as “*semaphorin receptor activity*”, “*axon guidance receptor activity*” or “*semaphorin-plexin signaling pathway involved in neuron projection guidance*” were particularly enriched in this dataset.

**Table 1:** GO term enrichment for the genes representative of the 3’174 proteins identified in the CSF dataset. GO term enrichment analysis was performed with Gorilla [6] on the three ontologies, (a) molecular function (b) biological process and (c) cellular component. The background used for the enrichment analysis was the full human proteome (UniProtKB/Swiss-Prot 2020/02 release). In the table, only terms with p-value below 10^−5^ and fold enrichment above 5, are displayed. All the enrichment results are presented in **Supplementary Tables S2-4**.

(a) Molecular Function				

GO number	GO term	Number of proteins identified in CSF	Total number of protein in human UniProtKB/Swiss-Prot	Fold enrichment

**GO:0097493**	structural molecule activity conferring elasticity	12	12	5.96
**GO:0048407**	platelet-derived growth factor binding	11	11	5.96
**GO:0030023**	extracellular matrix constituent conferring elasticity	10	10	5.96
**GO:0031995**	insulin-like growth factor II binding	8	8	5.96
**GO:0031994**	insulin-like growth factor I binding	12	13	5.51
**GO:0045499**	chemorepellent activity	23	25	5.49
**GO:0017154**	semaphorin receptor activity	11	12	5.47
**GO:0008046**	axon guidance receptor activity	8	9	5.30
**GO:0008191**	metalloendopeptidase inhibitor activity	14	16	5.22
**GO:0086080**	protein binding involved in heterotypic cell-cell adhesion	11	13	5.05

(b) Biological Process				

GO number	GO term	Number of proteins identified in CSF	Total number of protein in human UniProtKB/Swiss-Prot	Fold enrichment

**GO:0006957**	complement activation, alternative pathway	13	13	5.96
**GO:0097104**	postsynaptic membrane assembly	10	10	5.96
**GO:0048251**	elastic fiber assembly	9	9	5.96
**GO:0099545**	trans-synaptic signaling by trans-synaptic complex	8	8	5.96
**GO:1902669**	positive regulation of axon guidance	8	8	5.96
**GO:1902284**	neuron projection extension involved in neuron projection guidance	8	8	5.96
**GO:0048846**	axon extension involved in axon guidance	8	8	5.96
**GO:0061684**	chaperone-mediated autophagy	7	7	5.96
**GO:0048842**	positive regulation of axon extension involved in axon guidance	7	7	5.96
**GO:1902285**	semaphorin-plexin signaling pathway involved in neuron projection guidance	12	13	5.51
**GO:1902287**	semaphorin-plexin signaling pathway involved in axon guidance	11	12	5.47
**GO:0001941**	postsynaptic membrane organization	11	12	5.47
**GO:0042340**	keratan sulfate catabolic process	11	12	5.47
**GO:0097090**	presynaptic membrane organization	10	11	5.42
**GO:0097105**	presynaptic membrane assembly	9	10	5.37
**GO:0034371**	chylomicron remodeling	8	9	5.30
**GO:0071526**	semaphorin-plexin signaling pathway	31	35	5.28
**GO:0099560**	synaptic membrane adhesion	22	25	5.25
**GO:0042730**	fibrinolysis	19	22	5.15
**GO:0030207**	chondroitin sulfate catabolic process	12	14	5.11
**GO:0048841**	regulation of axon extension involved in axon guidance	26	31	5.00

(c) Cellular Component				

GO number	GO term	Number of proteins identified in CSF	Total number of protein in human UniProtKB/Swiss-Prot	Fold enrichment
**GO:0005577**	fibrinogen complex	8	8	5.96
**GO:0005593**	FACIT collagen trimer	7	7	5.96
**GO:0005579**	membrane attack complex	7	7	5.96
**GO:0005583**	fibrillar collagen trimer	11	12	5.47
**GO:0002116**	semaphorin receptor complex	10	11	5.42
**GO:0032279**	asymmetric synapse	8	9	5.30
**GO:0098651**	basement membrane collagen trimer	8	9	5.30
**GO:0042627**	chylomicron	11	13	5.05
**GO:0071682**	endocytic vesicle lumen	16	19	5.02

**Fig. 2:**
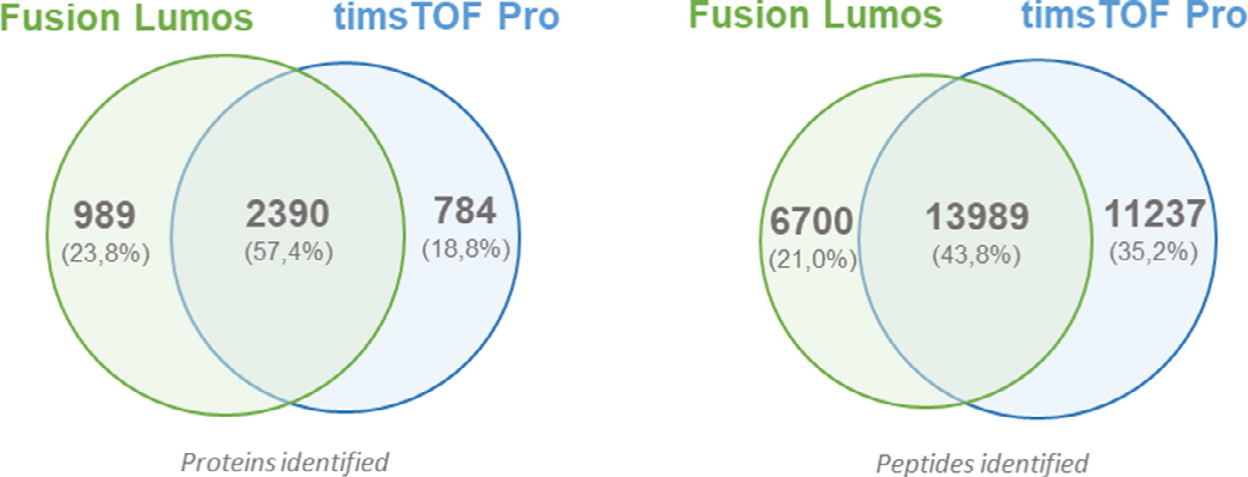
Comparison of protein and peptide identifications in CSF between our previously published dataset obtained with an Orbitrap Fusion Lumos instrument [1], and the current dataset obtained with a timsTOF Pro mass spectrometer.

When we compared this dataset to our previous data acquired with an Orbitrap Fusion Lumos instrument, identifying 20’689 peptides mapping on 3’379 proteins [1], we found that 57.4% of the proteins (*i.e.*, 2’390 proteins) were common to both datasets, as well as almost 14’000 peptides (*i.e.*, 43.8%) (Fig. 2).

## 2 Experimental design, materials, and methods

### 2.1 Sample preparation

The sample preparation was performed previously [1,5]. Briefly, 96 aliquots of 400 μL of a commercial pooled CSF sample (Analytical Biological Services) were evaporated with a *vacuum* centrifuge (Thermo Scientific). The dried samples were diluted in depletion Buffer A (Agilent Technologies) containing 9.65 µg/mL of β-lactoglobulin from bovine milk. Abundant CSF proteins were removed using MARS columns (Agilent Technologies) and HPLC systems (Thermo Scientific) equipped with an HTC-PAL (CTC Analytics AG) fraction collector. Buffer exchange was performed with Strata-X 33u polymeric reversed-phase (RP) (30 mg/1 mL) cartridges mounted on a 96-hole holder and a vacuum manifold, as previously described [7]. Samples were subsequently evaporated and subjected to reduction, alkylation, digestion, tandem mass tag (TMT) 6-plex (Thermo Scientific) labeling, pooling and purification using a 4-channels Microlab Star liquid handler workstation (Hamilton) in a 96-well-plate format and according to previously reported protocols [4,[7], [8], [9]]. Briefly, each sample was dissolved in 95 μL of triethylammonium bicarbonate (TEAB) 100 mM and 5 μL of 2% sodium dodecyl sulfate. A volume of 5.3 μL of tris(2-carboxyethyl) phosphine (20 mM) was added and incubation was performed for 1 h at 55°C. A volume of 5.5 μL of iodoacetamide 150 mM was added (incubation for 1 h in darkness). Enzymatic digestion was performed *via* the addition of 10 μL of trypsin/Lys-C at 0.25 μg/μL in 100 mM TEAB (incubation overnight at 37°C). TMT labeling was performed *via* the addition of 0.8 mg of TMT 6-plex reagent in 41 μL of CH_3_CN (incubation for 1 h at room temperature). After reaction, a volume of 8 μL of hydroxylamine 5% in H_2_O was added to each tube to react for 15 min. Samples from a given TMT 6-plex experiment were pooled together in a new tube. Pooled samples (*i.e.*, 16 pools in total from an original 96-samples set) were purified by solid phase extraction with Oasis HLB cartridges from Waters and Strata-X-C 33u polymeric strong cation cartridges from Phenomenex. All samples were resuspended in 200 μL of H_2_O/CH_3_CN/formic acid 96.9/3/0.1; 75 μL of the 16 resulting pooled samples were mixed together (to get enough material for sample fractionation), dried, and dissolved in 3232.8 µL H_2_O with 345.6 µL glycerol 50% and 21.6 µL of IPG buffer pH 3-10 (GE Healthcare Life Sciences). The sample was separated in 24 fractions with isoelectric focusing according to a previously published protocol [10], using the 3100 OFFGEL Fractionator (Agilent Technologies) and Immobiline DryStrip pH 3-10 (24 cm) (GE Healthcare Life Sciences).

### 2.2 RP-LC MS/MS analysis

The purified 24 fractions were dissolved in 50 µL H_2_O/CH_3_CN/formic acid (FA) 96.9/3/0.1%. A volume of 3 µL of each of the fractions were then diluted with 7 µL of H_2_O/FA 99.9/0.1% and only 2 µL of each diluted fraction were injected for separation on a 75 µm × 250 mm Aurora 2 C18 column (Ion Opticks). A typical RP gradient (Solvent A: 0.1% FA, 99.9% H_2_O MilliQ; Solvent B: 0.1% FA, 99.9% CH_3_CN) was run on a nanoflow LC system (nanoElute, Bruker Daltonik GmbH) at a flow rate of 400 nL/min. Column temperature was controlled at 50°C. The LC run lasted for 120 min (2% to 15% of Solvent B during 60 min; up to 25% at 90 min; up to 37% at 100 min; up to 95% at 110 min and finally 95% for 10 min to wash the column). The column was coupled online to a timsTOF Pro with a CaptiveSpray ion source (both from Bruker Daltonik GmbH). The temperature of the ion transfer capillary was set at 180°C. Ions were accumulated for 123 ms, and mobility separation was achieved by ramping the entrance potential from −160 V to −20 V within 123 ms.

The acquisition of mass and tandem mass spectra was done with average resolution of 60,000 and 50,000 full width at half maximum (mass range 100-1700 *m/z*), respectively. To enable the parallel accumulation-serial fragmentation (PASEF) method, precursor *m/z* and mobility information was first derived from full scan TIMS-MS experiments (with a mass range of *m/z* 100-1700). Singly charged precursors were excluded by their position in the *m/z*-ion mobility plane and precursors that reached a ‘target value’ of 20,000 a.u. were dynamically excluded for 0.4 min. The quadrupole isolation width was set to 2 Th for *m/z* < 700 and 3 Th for*m/z* ≥ 700, for fragmentation, and the collision energies varied between 31 and 52 eV depending on precursor mass and charge. TIMS, MS operation and PASEF were controlled and synchronized using the control instrument software OtofControl 5.1 (Bruker Daltonik). LC-MS/MS data were acquired using the PASEF method with a total cycle time of 1.23 s, including 1 TIMS MS scan and 10 PASEF MS/MS scans. The 10 PASEF scans (123 ms each) contained on average 12 MS/MS scans per PASEF scan. Ion mobility resolved mass spectra, nested ion mobility *versus m/z* distributions, as well as summed fragment ion intensities were extracted from the raw data file with DataAnalysis 5.1 (Bruker Daltonik).

### 2.3 Data processing and analysis

Protein identification was performed against the human UniProtKB/Swiss-Prot database (2020/02 release) comprising 20’367 protein sequences in total. Mascot (version 2.4.6 from Matrix Sciences) was used as search engine. Variable amino acid modifications were: oxidized methionine, deamidated asparagine/glutamine, and 6-plex TMT-labeled peptide amino terminus; 6-plex TMT-labeled lysine was set as fixed modifications as well as carbamidomethylation of cysteine. Trypsin was selected as the proteolytic enzyme, with a maximum of two potential missed cleavages. Peptide and fragment ion tolerance were set to 15 ppm and 0.05 Da, respectively. All Mascot result files were loaded into Scaffold Q+S 4.8.4 (Proteome Software) to be further searched with X! Tandem (The GPM, thegpm.org; version CYCLONE (2010.12.01.1)). The FDR in Scaffold was set up to 1% at protein and peptide level, with a one unique peptide criterion to report protein identification.

## Declaration of competing interest

The authors declare that they have no known competing financial interests or personal relationships which have, or could be perceived to have, influenced the work reported in this article. C. Macron, A. Núñez Galindo, M. Affolter and L. Dayon are employees of the Société des Produits Nestlé SA.

## Appendix Supplementary materials

Image, application 1

Image, application 2

## References

[1] Macron C., Lane L., Nunez Galindo A., Dayon L. (2018). Deep dive on the proteome of human cerebrospinal fluid: A valuable data resource for biomarker discovery and missing protein identification. J Proteome Res..

[2] Macron C., Nunez Galindo A., Cominetti O., Dayon L. (2019). A Versatile Workflow for Cerebrospinal Fluid Proteomic Analysis with Mass Spectrometry: A Matter of Choice between Deep Coverage and Sample Throughput. Methods Mol Biol.

[3] Nunez Galindo A., Macron C., Cominetti O., Dayon L. (2019). Analyzing Cerebrospinal Fluid Proteomes to Characterize Central Nervous System Disorders: A Highly Automated Mass Spectrometry-Based Pipeline for Biomarker Discovery. Methods Mol Biol.

[4] Nunez Galindo A., Kussmann M., Dayon L. (2015). Proteomics of cerebrospinal fluid: Throughput and robustness using a scalable automated analysis pipeline for biomarker discovery. Anal Chem.

[5] Macron C., Lane L., Nunez Galindo A., Dayon L. (2018). Identification of mssing proteins in normal human cerebrospinal fluid. J Proteome Res..

[6] Eden E., Navon R., Steinfeld I., Lipson D., Yakhini Z. (2009). GOrilla: a tool for discovery and visualization of enriched GO terms in ranked gene lists. BMC Bioinformatics.

[7] Dayon L., Nunez Galindo A., Corthesy J., Cominetti O., Kussmann M. (2014). Comprehensive and scalable highly automated MS-based proteomic workflow for clinical biomarker discovery in human plasma. J Proteome Res..

[8] Thompson A., Schafer J., Kuhn K., Kienle S., Schwarz J., Schmidt G., Neumann T., Johnstone R., Mohammed A.K., Hamon C. (2003). Tandem mass tags: a novel quantification strategy for comparative analysis of complex protein mixtures by MS/MS. Anal Chem.

[9] Dayon L., Hainard A., Licker V., Turck N., Kuhn K., Hochstrasser D.F., Burkhard P.R., Sanchez J.C. (2008). Relative quantification of proteins in human cerebrospinal fluids by MS/MS using 6-plex isobaric tags. Anal Chem.

[10] Dayon L., Sanchez J.C. (2012). Relative protein quantification by MS/MS using the tandem mass tag technology. Methods Mol Biol.

